# Induction of Innate Immune Memory by Engineered Nanoparticles: A Hypothesis That May Become True

**DOI:** 10.3389/fimmu.2017.00734

**Published:** 2017-06-26

**Authors:** Paola Italiani, Diana Boraschi

**Affiliations:** ^1^Institute of Protein Biochemistry, National Research Council, Napoli, Italy

**Keywords:** innate memory, monocytes, macrophages, engineered nanoparticles, inflammation

## Abstract

Innate immune memory is the capacity of cells of the innate immune system, such as monocytes and macrophages, to react differently to an inflammatory or infectious challenge if previously exposed to the same or to another agent. Innate immune memory is a protective mechanism, based on epigenetic reprogramming, that ensures effective protection while limiting side effects of tissue damage, by controlling innate/inflammatory responses to repeated stimulations. Engineered nanoparticles (NPs) are novel challenges for our innate immune system, and their ability to induce inflammatory activation, thereby posing health risks, is currently being investigated with controversial results. Besides their putative direct inflammation-inducing effects, we hypothesize that engineered NPs may induce innate memory based on their capacity to induce epigenetic modulation of gene expression. Preliminary results using non-toxic non-inflammatory gold NPs show that in fact NPs can induce memory by modulating in either positive or negative fashion the inflammatory activation of human monocytes to a subsequent bacterial challenge. The possibility of shaping innate/inflammatory reactivity with NPs could open the way to future novel approaches of preventive and therapeutic immunomodulation.

## Introduction

The ability of the body of developing immune reactions is strongly influenced by the environment. During its lifetime, each person is exposed to a great number and types of environmental and infectious cues, which shape the immune system in terms of type and extent of reaction. Consequently, the immune system of each individual is unique as it is the result of the individual experience. A recent study based on systems-level analysis of healthy twins has shown that different functional units of immunity (cytokines, chemokines, growth factors, immune cells subsets, and cellular responses to cytokines) vary across individuals primarily as a consequence of extrinsic non-heritable factors (1). This supports the notion that the immune system is shaped by the environmental events encountered during life (in particular microbes) rather than genetics. Environmental factors exert a cumulative influence that overshadows the influence of heritable traits with age (1). The footprints of these exposures are preserved in the immune cells, and each immune system can be considered as a kind of “memory snapshot/fingerprinting.” Consequently, the infection history of a person could explain the different individual patterns of immunodominance and protection and why some individuals mount productive immune responses to vaccines and pathogens and others do not. Until recently, the common belief was that adaptive immunity was the only type of immunity able to maintain a memory of previous infections. Indeed, in every immunology textbook we can find that memory is one of the hallmarks distinguishing adaptive from innate immunity. However, recent evidence has revived the old concept of innate immune memory, well-known in plants and invertebrates and also observed in mice.

Innate memory is the capacity of innate immune cells such as monocytes and macrophages to mount, upon a second challenge, a lower or higher non-specific response (tolerance vs. trained immunity) compared to the response of naïve cells (2). The innate response in usually measured in terms of production of inflammatory effector molecules (e.g., cytokines and chemokines). Thus, within each individual, innate immune cells such as monocytes are never the same and their reactivity depends on their immunological history of previous encounters and “the tracks that they left.” As long as they live, monocytes may display an altered responsiveness due to previous encounters not only with viral or bacterial infections but also following diseases and exposure to food/dietary components, pollutants, and nanoparticles (NPs).

**Figure 1:**
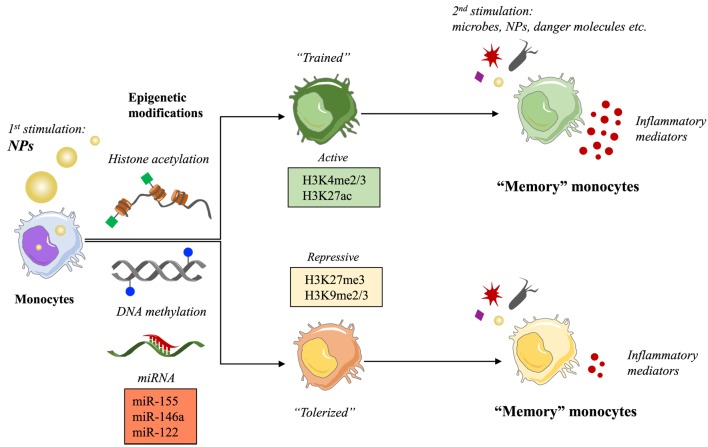
Nanoparticles (NPs) as possible inducers of innate immune memory. Schematic representation of the putative mechanism of innate memory induction by NPs.

Engineered NPs have entered the human environment in recent year because of their presence in many common products as additives (e.g., toothpaste, cosmetics, candies, and cigarettes) and in public spaces or workplaces as pollutants. The rapid development of nanotechnologies has also provided new opportunities in medicine mainly through the use of NPs for diagnostic and therapeutic purposes (biomedical imaging, drug delivery, and targeting). A lot of questions are still outstanding regarding the risks associated with exposure to NPs. Monocytes and macrophages are the first line of defense in the innate immune response to foreign materials, by phagocytosing and destroying the dangerous agents and in addition by triggering an inflammatory defensive reaction. An inflammatory reaction may, however, become pathological and lead to tissue destruction if it is excessive and prolonged (3). Over the last decade, a great deal of attention has been devoted to the study of the capacity of NPs to induce inflammation, taken as a sign of pathological risk. The inflammation-inducing effects of NPs are still controversial because of the many problems and challenges in the development and validation of assays that could reliably assess the *bona fide* NP effects, without interference and artifacts due to technical or contamination problems (4). Thus, many NPs do not show direct capacity of triggering an inflammatory reaction in human monocytes in culture when the interaction occurs in real life-mimicking conditions of dose and exposure, and if the NPs are rigorously free of contaminating LPS (bacterial endotoxin) (5, 6). Even if unable to directly initiate an inflammatory reaction, the exposure to NPs might interfere with the effector functions of monocytes and macrophages, including their activation, their polarization, and (as we propose here) their memory. For instance, it has been observed that NPs can provoke morphological changes, proliferation alterations, toxicity, functional phenotype switching, and epigenetic reprogramming (7–11). To date, the epigenetic reprogramming is known to be the main mechanism underlying the capacity of innate immune cells to develop a memory. Here, we would like to discuss the possible influence of NPs on the development of innate memory, in other words if previous exposure to NPs can modulate the responses of monocytes and macrophages to subsequent infections or challenges (Figure 1).

## Innate Memory and Underlying Epigenetic Mechanisms

The immune system has evolved with the increased complexity of living organisms (innate immunity only in plants and invertebrates; innate plus adaptive immunity in vertebrates). More impressively, immunity developed in parallel with the evolution of microorganisms in an equilibrium in which the host develops tools for keeping the microorganisms at bay and avoid damage, and the pathogen devises mechanisms for escaping the host surveillance and ensure its own survival and growth (12). The ability of the adaptive immune system to recognizing different challenges is mainly due to the rearrangement of V(D)J gene segments, aimed to generating a vast array of different specific antibodies and receptors necessary for the recognition of virtually all non-self-molecules, which are then conserved by B and T memory cells throughout lifetime. As a consequence, one of the most potent weapons of adaptive immunity is to implement a faster and more potent defense response upon a second exposure to the same pathogen, due to the ability to “remember” a first encounter. This capacity to remember, considered a distinctive trait of adaptive immunity, can be, however, found also in vertebrate innate immunity, although with different characteristics. Innate memory is already well-known as the protective mechanism against reinfection in organisms lacking adaptive immunity, such as plants and invertebrates (13, 14). Thus, the dogma that innate immunity has no memory should be revised, as the capacity of memory has been described in innate cells belonging to both the lymphoid lineage, such as natural killer (NK) cells, and to myeloid cells such as monocytes and macrophages. For instance, upon human cytomegalovirus infection in mice and macaques, certain NK cell subpopulations display adaptive properties such as longevity, subset expansion, and altered functionality during a secondary response (15). The main differences between innate and adaptive immune memory are summarized in Table 1.

**Table 1:** The main differences between innate and adaptive immune memory.

	Innate memory	Adaptive memory
Effector molecules	Cytokines	Antibodies
Mechanisms	Epigenetic changes (e.g., DNA methylation, histone acetylation)	Gene rearrangement (somatic recombination of gene segments)
Type of response	Rapid (same as primary response), either enhanced (“trained memory”) or reduced (“tolerance”)	Rapid (much more than primary response), enhanced/more potent
Specificity	Triggered by any molecule or stressful event (e.g., molecules shared by groups of related microbes or produced by damaged host cells, metabolic compounds, pollutants, etc.), upon a second exposure to the same or different agent/event	For a specific antigen, upon a second exposure to the same

Focusing our attention on monocytes and macrophages, the innate immune memory appears as an increase (“trained immunity”) or a decrease (“tolerance”) of their functional program. Thus, primed monocytes or macrophages become more or less capable of producing inflammatory cytokines, as well as phagocytosing and killing microorganisms, in response to a second challenge. It is hypothesized that this altered functional state could persist for weeks to months, rather than years, after the elimination of the initial stimulus (16), although it might persist much longer in bone marrow niches. The main difference between innate and adaptive memory is that innate memory is non-specific. Although this could seem a limitation, it has the advantage of protecting against different kinds of inflammation-inducing challenges, not only microbes and the same microbes. To put it simply, primed monocytes can react or not following a secondary challenge, which can be the same or different from the primary stimulus, conferring a non-specific and broad protection.

The phenomenon of tolerance upon chronic or repeated exposure to microbial agents is well-known and represents a state of refractoriness to additional challenge with microbial molecules such as LPS (17, 18). Tolerance has also been identified as the hyporesponsiveness/immunosuppressive phenotype observed in late sepsis. Indeed, tolerance is viewed as a defense strategy to limit inflammation-caused tissue damage (19). Conversely, the newer concept of “trained immunity” arises from a number of epidemiological studies that suggest non-specific beneficial effects of vaccination beyond its target disease (20). For instance, one of the world’s most administrated vaccines, the Bacille Calmette–Guérin (BCG), protects not only against tuberculosis but it also has positive effects on neonatal sepsis and respiratory tract infections (21), and improves resistance and survival of infants (22). These observations have been confirmed by experimental studies in murine models lacking T and B lymphocytes, which proved that BCG vaccination has non-specific effects against pathogens other than mycobacteria, such as *Candida albicans*. In turn, administration of *C. albicans* protects against infection by a number of different bacteria (23) as well as against itself (24). Recent studies proved that human monocytes stimulated *in vitro* with *C. albicans* β-glucan or from subjects vaccinated with BCG have increased capacity to produce inflammatory cytokines, as well as to phagocytosing and killing microorganisms (16, 24).

Regarding the molecular mechanisms involved in the development of the innate memory, studies on Systemic Acquired Resistance in plants showed that epigenetic processes are responsible for the resistance to reinfection (13). Other studies have demonstrated that regulation of chromatin states is on the basis of the innate immune tolerance induced by LPS (25). Indeed, both tolerance and trained innate immunity in monocytes and macrophages are dependent on long-term epigenetic changes. These modifications involve both histone methylation and acetylation, such as H3K4 monomethylation and H3K27 acetylation induced by LPS (26, 27), and histone H3K4 trimethylation and H3K27 acetylation caused by β-glucan (24, 27). Moreover, it has been hypothesized that innate memory could involve the modulation of expression of “latent and *de novo*” enhancers, microRNAs, and/or long non-coding RNAs (28). All these epigenetic changes and molecular mechanisms promote higher transcription levels in several genes, such as pathogen recognition receptors, signaling molecules, and cytokines (24), in a short window of time. An accurate description of all these mechanisms and the role of cellular metabolites in shaping the epigenetic program of innate immune memory has been recently published in an excellent review (28).

## The Effects of NPs on the Epigenome

Monocytes and macrophages are not only activated by microorganisms but they can react to any harmful stimulus by initiating an inflammatory reaction. Accordingly, all these agents might prime monocytes and macrophages and reprogram their reactivity against a subsequent stimulation, i.e., they can induce innate memory.

In the last few years, our immune system has become exposed to a new class of agents, i.e., the engineered nanomaterials, which have entered our life because of their successful use in many products, thanks to their physical and chemical properties (size, chemical composition, surface properties, solubility, shape, etc.). Apart from the advantages of such new materials, the possible detrimental effects of exposure to NPs are being actively investigated from a safety point of view. Being the innate immune system the first line of defense of the body, and monocytes and macrophages among the first cells which NPs interact with, assessing the outcomes of such interaction becomes a priority in order to avoid harmful effects that can damage tissues and organs of the body (induction of uncontrolled inflammation) both in the case of NPs for medical use and in the case of occasional or unintentional exposure. Also, knowing the ways of nanoimmune interaction can help us avoiding the immune-mediated rapid elimination of nanomedicines that are detected as possible dangers by the immune system. The consequences of the interaction between NPs and immune system have been extensively discussed (29). Here, we want to focus on the effects of NPs on the epigenome. It is known that the gene expression pattern of a cell is modulated (upregulated or silenced) by epigenetic changes, such as DNA methylation, post-translational modifications of histones, chromatin remodeling, and modulation of non-coding RNAs. Several NPs have shown the capacity of inducing epigenetic effects, which may alter gene expression and in the long run may lead to health risks. For instance, a decrease in global DNA methylation has been observed in human epidermal keratinocytes following exposure to SiO_2_ NPs *in vitro* and in the lungs and blood of mice upon inhalation of multiwall carbon nanotubes (30, 31). Regarding the effect of NPs on histone post-translational modifications, little is known so far. A preliminary study showed that exposure to cadmium telluride quantum dots induced global H3 histone hypoacetylation and reduced gene transcription in a breast cancer cell line (32). In another study, silver NPs induced a decrease in methylation of H3K4me3 and H3K79me1 in mouse erythroleukemia cells, causing a reduction in hemoglobin levels (33). NPs can also affect ncRNAs.

The effects of NPs on miRNA expression have been observed both *in vivo* and *in vitro*. Inhaled surface-coated nanoTiO_2_ and intravenous doses of silica NPs resulted in an enrichment of miRNA expression in mouse lung (miR-1, miR-49a, and miR-135b) and liver (miR-122), respectively (34, 35). *In vitro* exposure to gold NPs upregulated the expression of miR-155 in human fetal fibroblasts (36), and exposure of the human Jurkat T cell line to silver NPs altered the expression of 63 miRNAs (37). A high-throughput sequencing analysis of a mouse fibroblast cell line exposed to iron oxide, quantum dots, and carbon nanotubes resulted in widely dysregulated miRNA expression profiles depending on the characteristic of nanomaterials (38).

All the known effects of NPs on the epigenome have been recently reviewed in detail elsewhere (9–11). However, the consequences of such changes on cellular functions and the eventual impact on human health are far from being known.

## Could NPs Affect/Modulate the Innate Immune Memory?

**Figure 2:**
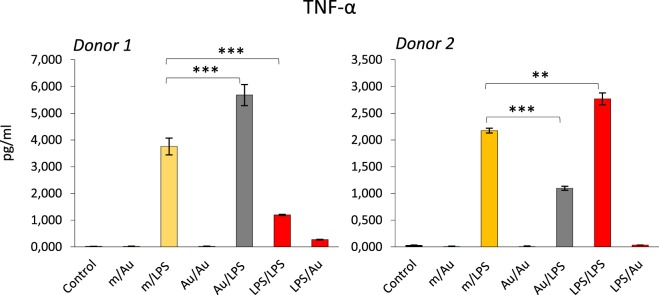
Modulation of innate memory by Au nanoparticles (NPs). Freshly isolated human monocytes were exposed to medium alone (m) or containing bacterial LPS (LPS) (0.1 ng/ml) or Au NPs (Au) [40 nm, provided by Prof. Victor F. Puntes, ICN2, Barcelona; 10 ng/ml (4, 5)] for 24 h (priming). After elimination of the stimuli, cells were rested for 6 days, and then challenged for 24 h with either LPS (1 ng/ml) or Au NPs (10 ng/ml). Controls are cells primed with medium, LPS, or Au NPs and challenged with medium alone (m/m, LPS/m, Au/m; control) and were all negative. Production of TNF-α after challenge was measured by ELISA. Data from two different donors are shown. Priming with Au NPs increased the response to an LPS challenge compared to unprimed cells in donor 1, whereas a decrease was observed in donor 2. Conversely, LPS priming decreased the response to an LPS challenge in donor 1 and increased it in donor 2. The characteristics of the Au NPs used in this study are reported in Ref. (5). The contamination with LPS (endotoxin) was assessed by the limulus amebocyte lysate assay and found to be <0.005 EU/μg particles (41). Student’s *t*-test was used to analyze statistically significant differences. The differences between controls and treatments are all statistically significant, but the *p*-value is not indicated to avoid overwriting the figure. We indicated only the differences discussed in the text. **p < 0.01, ***p < 0.001.

Since an epigenetic reprogramming is the major molecular mechanism underlying the establishment of innate memory, and the NP exposure could alter the epigenetic program in monocyte-like cell lines (39, 40), it is logical to hypothesize that NPs may be able to induce or modulate innate memory, and therefore, affect the capacity of innate cells to react to dangerous stimuli. The hypothesis that NPs can modulate innate memory adds a new perspective in the evaluation of nanoimmune interactions in terms of functional outputs, both from the point of view of safety and, most interestingly, for its possible medical exploitation in reprogramming innate memory in immunostimulatory and immunosuppressive strategies (vaccination, age- or disease-related immunosuppression, chronic inflammatory and degenerative diseases, etc.). As proof-of-concept, we have preliminarily assessed the role of gold (Au) NPs in the induction of innate memory in an *in vitro* system based on human primary monocytes. Monocytes were incubated with LPS or with endotoxin-free Au NPs for 24 h (priming), then rested for 6 days in the absence of stimuli, and eventually restimulated (challenge) with the same stimulus or cross-stimulated with the other agent. Figure 2 shows preliminary data obtained by measuring the production of the inflammatory cytokine TNF-α by monocytes from two individual donors. It is important to say (not shown in the figure) that in response to the first stimulation LPS induced a significant response while Au NPs were completely inactive. After 6 days of resting, all cells were fully rested, i.e., they did not produce any measurable amount of the cytokine (not shown). When challenged with LPS, cells primed with LPS showed either a tolerant or a trained response, depending on the donor. When primed with Au NPs, an opposite response to LPS was observed, i.e., cells that were tolerant when primed with LPS were trained if primed with Au NPs and *vice versa*. Notably, not only naïve cells but also primed cells (either with LPS or with Au NPs) could not be stimulated by Au NPs to produce TNF-α. Several important considerations arise from these observations. The first is that NPs, even when unable to directly activate monocytes, could induce a memory that modulates the cell reactivity to a subsequent challenge. The second consideration is that, as expected, each individual subject responds differently not only in quantitative terms (the amount of cytokine produced) but also in terms of type of response (enhanced reaction vs. decreased response). This behavior most likely depends on the past history of exposure of the donor, i.e., age, vaccinations, diseases, etc. Moreover, it is important to note that, since the same stimulus is able to prime for decreased or increased responses in different donors, the innate memory seems to be a complete reprograming of the reactivity of cells rather than a stimulus-dependent inhibition or enhancement, a reprogramming that, again, most likely depends on the past “history” of the monocytes of each individual.

## Concluding Remarks and Future Perspectives

Although the study of the effect of NPs on human epigenome is still in its infancy, it is possible to speculate that NPs, like all other foreign agents that come in contact with the innate immune system, have the potential of modulating the innate memory in monocytes and macrophages through epigenetic changes. Plants and animals, including human beings, live in an environment that constantly expose them to challenges, including an enormous variety of microorganisms and other parasites, in addition to chemical compounds, pollutants, NPs, and many others. All the agents that confront the innate immune cells can prime them, so that these cells are more ready to mount an adequate protective response upon subsequent challenges. This is a general protective mechanism that aims at maintaining a good protective response while avoiding, in particular in situation of frequent exposures, excessive damage to the body (as in the case of endotoxin tolerance). Examining the effects of engineered NPs in this context is of great importance. We have seen that innocuous NPs such as Au NPs can induce memory and change the response of monocytes to bacterial compounds (represented by LPS). This means that NPs are in fact behaving like microbial agents in terms of ability to induce innate memory and consequent reprogramming of innate reactivity. The effect of NPs on innate memory, as in the case of microbial compounds, depends both on the physicochemical nature of the NP and on the history of previous exposure of the subject. Thus, NP safety and efficacy studies would need to consider a personalized approach, because we expect each subject to respond differently both from others and in different periods of his/her life. From this perspective, it is exciting that the hypothesis that the manipulation of innate memory with NPs may become an effective immunomodulatory therapeutic option in future approaches of precision medicine.

## Author Contributions

PI wrote the article and drew the figures; DB critically revised it.

## Conflict of Interest Statement

The authors have no financial or non-financial competing interests with the content of the article.
